# People with Diabetes Have Poorer Self-Rated Health (SRH) and Diabetes Moderates the Association between Age and SRH

**DOI:** 10.3390/diseases11020073

**Published:** 2023-05-12

**Authors:** Weixi Kang, Antonio Malvaso

**Affiliations:** 1 UK DRI Care Research and Technology Centre, Department of Brain Sciences, Imperial College London, London W12 0BZ, UK; 2 IRCCS “C. Mondino” Foundation, National Neurological Institute, Department of Brain and Behavioral Sciences, University of Pavia, 27100 Pavia, Italy

**Keywords:** diabetes, age, self-rated health

## Abstract

Diabetes is a severe chronic condition that is related to decreased physical functioning. Recently, there has been growing interest in understanding how a brief report on health such as self-rated health (SRH) could be used to track changes in health status and service needs in people with diabetes. The current research aims to investigate how SRH is affected by diabetes and how diabetes could moderate the association between age and SRH. By analyzing data from 47,507 participants, with 2869 of them clinically diagnosed with diabetes, the current study found that people with diabetes had significantly poorer SRH than people without diabetes after controlling for demographic covariates (t(2868) = −45.73, *p* < 0.001, 95% C.I. (−0.92, −0.85), Cohen’s d = −0.85). In addition, diabetes was a significant moderator of the relationship between age and SRH (*b* = 0.01, *p* < 0.001, 95% C.I. (0.01, 0.01)). Specifically, age was more strongly related to SRH in people without diabetes (*b* = −0.015, *p* < 0.001, 95% C.I. (−0.016, −0.015)) than in people with diabetes (*b* = −0.007, *p* < 0.001, 95% C.I. (−0.010, −0.004)). Health professionals should aim to improve SRH in people with diabetes given that SRH is related to various outcomes.

## 1. Introduction

Diabetes is a severe chronic condition that is related to decreased physical functioning [1]. Recently, there has been growing interest in understanding how a brief report of health could be used to track changes in health status and service needs in people with diabetes, which could provide information on health in a population over time and enable decisions regarding public health actions to be made [2]. Moreover, these kinds of measures can be carried as a measure of disparities within countries and as comparisons across countries [3].

Self-rated health (SRH) has gained attention as a valuable tool in population health research as it captures an individual’s subjective perception of their overall health status [2]. While SRH is not a disease-specific measure, it has been shown to have predictive validity for various health conditions, including lung disease, arthritis, functional stroke impairments, cardiovascular disease, depression, and mortality [4,5]. One of the main strengths of SRH is its ability to capture the net effect of various risk factors, including unmeasured health risk factors, on an individual’s health status [6]. In addition to capturing the impact of health risk factors, SRH has been linked to lifestyle factors, such as smoking, alcohol consumption, physical activity, unhealthy diet, and obesity, which have been identified as risk factors for diabetes [7,8,9]. Furthermore, studies have shown that SRH is strongly associated with objective health measures and declines with age, making it a useful indicator of health status in older populations [10]. Elevated inflammatory markers have also been linked to a negative relationship with SRH, which is a risk factor for diabetes [11,12,13]. Therefore, incorporating SRH as a measure of overall health status may provide valuable information for public health interventions and enable the monitoring of health status changes in populations over time. Recent studies have highlighted the importance of incorporating SRH in diabetes management, as it can provide insight into the complex interplay between diabetes, comorbidities, and lifestyle factors that impact overall health outcomes [14,15].

Several cross-sectional studies have also found a negative relationship between SRH and diabetes [16,17]. Moreover, two previous studies have also shown that a lower score in SRH is associated with a higher risk of incident diabetes [18,19,20]. Specifically, Wennberg et al. [21] found that type 2 diabetes risk was increased in people with lower SRH. The biggest health-related factor, obesity, could only partially account for the link. They discovered no evidence of variability in the relationship between type 2 diabetes mellitus and SRH across the European centers [19]. Recently, among 729 African-American male participants in the 2017 and 2018 African-American Male Wellness Walks, the relationship between a combination of ideal cardiovascular health (ICH) metrics (blood pressure, glucose, cholesterol, body mass index (BMI), physical activity, and smoking) and SRH, diabetes, and body fat percentage was investigated [21]. For African-American men, greater ICH scores are linked to better SRH, a decreased risk of diabetes, and a lower body fat percentage [21].

Although studies have shown that SRH strongly correlates with objective health [22], and changes over time given health generally declines with age [2], much less is known about the trajectory of SRH in people with chronic conditions such as diabetes. One study found that the SRH trajectory of most people with diabetes tends to increase within a four-year period [23]. However, these types of studies only looked at SRH in a four-year period, and much less is known about whether diabetes can moderate the association between age and SRH in people from all age groups. Thus, the current study aimed to investigate how SRH is affected by diabetes and how diabetes could moderate the connection between age and SRH. The results of the current study will provide a novel understanding about how diabetes could modify the associations between age and SRH, which is important for outcomes, such as morbidity and mortality.

## 2. Methods

The present study is based on data collected from the Understanding Society survey, which is a nationally representative longitudinal survey of households in the UK. The dataset used in this study is publicly available at https://www.understandingsociety.ac.uk, accessed on 22 October 2022. All data collections were approved by the University of Essex Ethical Committees, and informed consent was obtained from all participants. The present study used data from Wave 1 of the survey, which was conducted between 2009 and 2010 [24]. The sample included 47,507 participants, among whom 2869 were clinically diagnosed with diabetes. The large sample size and nationally representative nature of the Understanding Society survey make it a valuable resource for investigating health outcomes in the UK population.

People with clinically diagnosed diabetes also answered a question regarding if they have been clinically diagnosed with diabetes: “Has a doctor or other health professional ever told you that you have any of these conditions? Diabetes”. Self-reported diabetes is a valid measure of diabetes status (e.g., [25]). Participants also responded to the question, “In general, would you say your health is …” using a 5-point scale ranging from 1 (excellent) to 5 (very poor). The reliability of this single measurement of subjective health is moderate (e.g., [26]). SRH was reverse-coded, so a higher score reflects a better SRH. Demographic variables include age, sex, monthly income, highest educational qualification, and present marital status.

In this study, all statistical analyses were conducted using MATLAB 2018a, a high-level programming language commonly used for scientific computing. A predictive normative modeling approach was utilized to investigate differences in SRH between individuals with and without diabetes. A generalized linear model was constructed in MATLAB 2018a to predict SRH based on demographic variables, with SRH being the predicted variable and demographic variables as predictors. This model was then used to estimate the expected SRH for people with diabetes, given their demographic characteristics. To determine if there was a significant difference between predicted SRH and actual SRH for people with diabetes, a one-sample t-test was conducted. This statistical approach enabled us to assess the impact of diabetes on SRH while controlling for demographic variables.

In order to examine the relationship between age and SRH among individuals with and without diabetes, and whether diabetes modifies the trajectory of SRH with age, we employed a hierarchical regression approach, with age, sex, monthly income, highest educational qualification, present legal marital status, and the interaction of age and diabetes status included as predictors to estimate the effect of age on SRH. Two separate multiple regressions were then performed to examine the association between age and SRH among individuals with and without diabetes, respectively. By comparing the coefficients from the two models, we can determine if the strength and direction of the relationship between age and SRH differ for individuals with and without diabetes.

## 3. Results

The descriptive statistics can be found in Table 1. In the generalized linear model trained on people without diabetes, the current study found a significant main effect of age (F(1, 44,632) = 2564.30, *p* < 0.001), sex (F(1, 44,632) = 4.69, *p* < 0.05), monthly income (F(1, 44,632) = 251.03, *p* < 0.001), highest educational qualification (F(1, 44,632) = 714.99, *p* < 0.001), and present legal marital status (F(1, 44,632) = 250.62, *p* < 0.001) on SRH. The main finding was that people with diabetes had significantly poorer SRH than people without diabetes after controlling for demographic covariates (t(2868) = −45.73, *p* < 0.001, 95% C.I. (−0.92, −0.85), Cohen’s d = −0.85).

**Figure 1:**
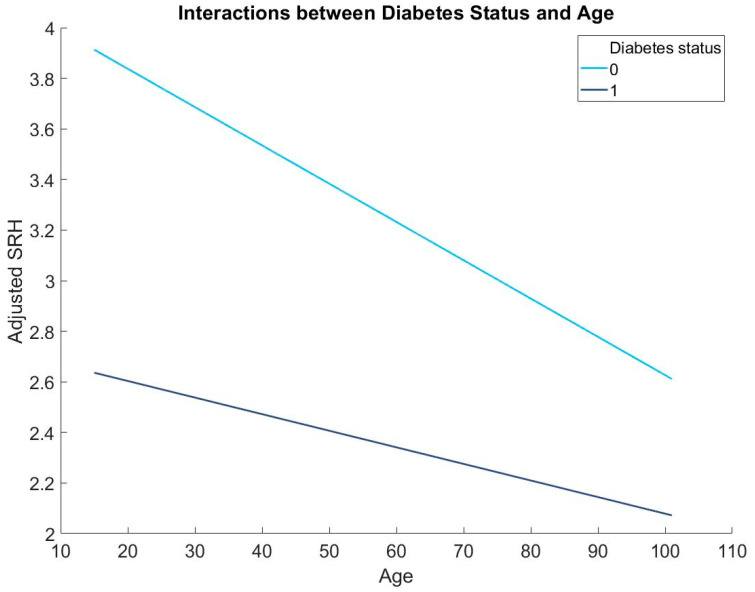
The moderating role of diabetes in the association between age and SRH: 0 = people without diabetes and 1 = people with diabetes.

In addition, results from the hierarchical regression revealed that diabetes status is a significant moderator in the association between age and SRH (*b* = 0.01, *p* < 0.001, 95% C.I. (0.01, 0.01); Table 2; Figure 1). Simple slope regressions showed that age is more strongly associated with SRH in people without diabetes (*b* = −0.015, *p* < 0.001, 95% C.I. (−0.016, −0.015)) than people with diabetes (*b* = −0.007, *p* < 0.001, 95% C.I. (−0.010, −0.004)). 

## 4. Discussion

The aim of the current study was to test how SRH is affected by diabetes and examine how diabetes could modify the connection between age and SRH. Findings from the current study provided novel findings regarding that SRH is negatively affected by diabetes and diabetes status is a significant moderator in the association between age and SRH. Specifically, the negative association between age and SRH was stronger in people without diabetes compared to people with diabetes.

The finding that people with diabetes have poorer SRH is largely consistent with previous studies that found a negative relationship between SRH and diabetes [16,17,18,19]. People with diabetes may have poor SRH due to a number of factors. Firstly, diabetes requires continuous self-care, which can be physically and emotionally demanding and can take a toll on an individual’s mental and emotional well-being [27,28,29]. Secondly, diabetes increases the risk of developing other health complications, which can further exacerbate an individual’s health status and lead to a decline in SRH [27]. Thirdly, diabetes can affect an individual’s quality of life by limiting their ability to participate in daily activities, causing social isolation and leading to feelings of anxiety and depression, all of which can contribute to a negative self-perception of one’s health status [28,30]. Fourthly, this association may be explained by the relationship between SRH and inflammatory markers, which is a risk factor for diabetes [11,12,13]. Thus, this association could be bi-directional. Future studies should test if elevated inflammatory markers mediate the relationship between diabetes and SRH. Finally, the brain being affected by diabetes [31,32] and the use of anti-hyperglycemic drugs may then lead to poor SRH. Overall, the complex and multifaceted nature of diabetes and its associated complications can lead to poor SRH among individuals with diabetes.

The current study also found that age is negatively associated with SRH in people with and without diabetes, which was largely consistent with previous studies [2]. However, this association in people with diabetes was much weaker than people without diabetes, which may be explained by the fact that people with diabetes already had much poorer SRH compared to people without diabetes; thus, age was no longer that sensitive to people with diabetes. Alternatively, it could be that the impact of age on SRH in people with diabetes is obscured by other factors, such as the severity and duration of the diabetes, comorbidities, and treatment regimens [33]. These factors could also contribute to the weaker association between age and SRH in people with diabetes.

There are some limitations in the current study. First, the type of diabetes that participants had (e.g., type 1 vs. type 2) remains unclear, and type 1 and type 2 diabetes may have very different effects on SRH. Future studies should investigate the effect of specific types of diabetes on SRH. Second, all the measures were self-reported, so self-reporting bias could not be avoided. Third, the cross-sectional nature of the current study made it impossible to establish causality. This study implies that SRH measures may be effective indicators of health status in diabetes patients. Finally, since diabetes is a complex disease, which affects almost all the organs of the body, in particular, the brain, the SRH might be due to uncontrolled diabetes, leading to damage to the brain. However, we did not have information regarding whether diabetes was controlled, moderately controlled, and uncontrolled. Effective diabetes management requires a multifaceted approach, including lifestyle modifications, pharmacological interventions, and regular monitoring of blood glucose levels. Lifestyle modifications, such as regular physical activity and a healthy diet, are essential components of diabetes management. Pharmacological interventions, including insulin and oral hypoglycemic agents, are also commonly used to control blood glucose levels. Additionally, regular monitoring of blood glucose levels is crucial for adjusting treatment regimens and preventing diabetes-related complications. Overall, the management of diabetes requires a comprehensive and individualized approach tailored to each patient’s unique needs and circumstances.

## 5. Conclusions

Taken together, the paper explored the use of SRH as a valuable tool in tracking changes in health status and service needs in people with diabetes. The study found that people with diabetes have a lower SRH compared to those without diabetes, and age moderates the relationship between diabetes and SRH. The results suggest that diabetes could have a negative impact on SRH, especially at older ages. The study may imply that incorporating SRH as a measure of overall health status could provide valuable information for public health interventions and enable the monitoring of health status changes in populations over time. The findings also highlight the importance of the early detection and management of diabetes to improve health outcomes and quality of life for people with diabetes. Health professionals should aim to improve SRH in people with diabetes given that SRH is related to various outcomes. Specifically, social participation [34], e-Health tools [35], and physical activities [36] should be encouraged as they are related to better SRH. However, the study has some limitations, including the use of a single measurement of SRH, the reliance on self-reported diabetes status, and the cross-sectional design, which limits causal inference. Future research should consider a longitudinal design and objective measures of health to better understand the trajectory of SRH in people with diabetes.

## Figures and Tables

**Table 1:** Descriptive statistics of demographic characteristics, diabetes status, and SRH.

Variables	Mean	S.D.
Age (year)	45.94 (15–101)	18.14
Monthly income (£)	1219.67 (0–47, 442.9)	1327.57
SRH	3.39 (1–5)	1.15
	N	%
**Sex**		
Male	20,913	44.02
Female	26,594	55.98
**Highest educational qualification**		
Below college	34,245	72.08
College	13,262	27.92
**Present legal marital status**		
Single	23,433	49.33
Married	24,074	50.67
**Diabetes status**		
Yes	2869	6.04
No	44,638	93.96

**Table 2:** The regression coefficient (*b*) for demographics, diabetes status, and age by diabetes status interactions with the total explained variances (R2). All numbers were rounded up to two digits. Effect sizes between 0.10 and 0.29 are said to be only small, effect sizes between 0.30 and 0.49 are medium, and effect sizes of 0.50 or greater are large.

Variables	b	SE	P	95% C.I.
Age	−0.02 ***	0.00	0.03	[−0.02, −0.01]
Sex	−0.02 ***	0.01	0.00	[−0.04, 0.00]
Monthly income	0.00 ***	0.00	0.00	[0.00, 0.00]
Highest educational qualification	0.31 ***	0.01	0.00	[0.29, 0.33]
Marital status	0.17 ***	0.01	0.00	[0.15, 0.19]
Diabetes status	−1.41 ***	0.08	0.00	[−1.57, −1.24]
Diabetes status: Age	0.01	0.00	0.00	[0.01, 0.01]
R2	0.14			

*** *p* < 0.001.

## Data Availability

Publicly available datasets were analyzed in this study. These data can be found here: https://www.understandingsociety.ac.uk (accessed on 7 February 2022).
